# Supplementary material for: Embedding Biomimetic Magnetic Nanoparticles Coupled with Peptide AS-48 into PLGA to Treat Intracellular Pathogens
Source: Pharmaceutics. 2022 Dec 8;14(12):2744. doi: 10.3390/pharmaceutics14122744 (PMC9785849; doi:10.3390/pharmaceutics14122744)
Supplement: Supplementary file 1 [file pharmaceutics-14-02744-s001.zip › pharmaceutics-2002062-supplementary.pdf]

pharmaceutics

MDPI

Article

Embedding biomimetic magnetic nanoparticles, coupled with AS-48 peptide, into PLGA for the treatment of intracellular pathogens

Salvatore Calogero Gaglio[1†], Ylenia Jabalera[2†] Manuel Montalbán-López[2], Ana C. Millán-Placer[3], Marina Lázaro-Callejón[5,] Mercedes Maqueda[2], María Paz Carrasco Jimenez[4], Alejandro Laso[4], José A. Aínsa[3], Guillermo R. Iglesias[5], Massimiliano Perduca[1*] and Concepcion Jimenez-Lopez[2*]

1. Department of Biotechnology, University of Verona, Strada Le Grazie 15, 37134 Verona, Italy
2. Department of Microbiology, Faculty of Sciences, University of Granada, 18071 Granada, Spain
3. Departamento de Microbiología, Pediatría, Radiología y Salud Publica (Facultad de Medicina) & BIFI, Universidad de Zaragoza, 50009 Zaragoza, Spain; CIBER de Enfermedades Respiratorias (CIBERES), Instituto de Salud Carlos III, Madrid, Spain
4. Department of Biochemistry and Molecular Biology I, University of Granada, 18071 Granada
5. Department of Applied Physics and Instituto de Investigación Biosanitaria ibs.GRANADA, NanoMag Laboratory, University of Granada, 18071 Granada, Spain

* Correspondence: cjl@ugr.es (C.J.-L.); massimiliano.perduca@univr.it (M.P.)

† These authors equally contributed

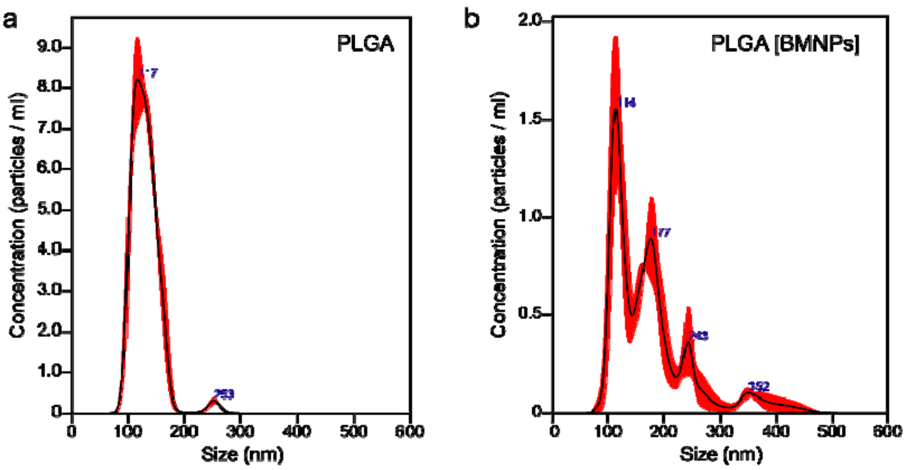

Figure S1. NTA analysis of different control samples: (a) PLGA and (b) PLGA[BMNPs].

Table S1. Summary of specific absorption rate (SAR) and intrinsic loss power (ILP) calculations concerning PLGA(AS-48-BMNPs) at different intensities and at fixed frequency of 120 kHz, after 30 seconds of exposure. Sample volume 0.2 ml.

Nano-formulation	Field (kA/m)	SAR (W/g)	ILP (nHm^2kg^{-1})
BMNPs	13.4 ± 0.2	373 ± 29	20.0 ±1.5
	17.7 ± 0.2	595 ± 40	18.7 ± 1.2
	23.2 ± 0.2	1210 ± 130	20.8 ± 2.1

Nano-formulation	Field (kA/m)	SAR (W/g)	ILP (nHm^2kg^{-1})
PLGA[BMNPs]	13.4 ± 0.2	252 ± 23	13.6 ± 1.2
	17.7 ± 0.2	416 ± 30	13.1 ± 1.0
	23.2 ± 0.2	760 ± 50	13.0 ± 0.9

Nano-formulation	Field (kA/m)	SAR (W/g)	ILP (nHm^2kg^{-1})
AS-48-BMNPs	13.4 ± 0.2	193 ± 12	10.4 ± 0.7
	17.7 ± 0.2	322 ± 30	11.6 ± 1.9
	23.2 ± 0.2	625 ± 30	10.7 ± 0.6

Nano-formulation	Field (kA/m)	SAR (W/g)	ILP (nHm^2kg^{-1})
PLGA[AS-48-BMNPs]	13.4 ± 0.2	314 ± 21	16.9 ± 1.1
	17.7 ± 0.2	440 ± 40	13.8 ± 1.1
	23.2 ± 0.2	836 ± 100	14.4 ± 1.7

Table S2. Summary of the calculation of the specific absorption rate (SAR) for all nano-formulations at different laser power densities after 30 seconds of exposure. Sample volume 0.2 ml

Nano-formulation	Power (W/cm^2)	SAR (W/g)
BMNPs	0.5 ± 0.1	13.5 ± 1.3
	1.0 ± 0.1	37.1 ± 1.3
	2.0 ± 0.1	70.4 ± 2.9

Nano-formulation	Power (W/cm^2)	SAR (W/g)
PLGA[BMNPs]	0.5 ± 0.1	14.9 ± 1.5
	1.0 ± 0.1	36.9 ± 1.3
	2.0 ± 0.1	80.7 ± 1.5

Nano-formulation	Power (W/cm^2)	SAR (W/g)
AS-48-BMNPs	0.5 ± 0.1	18.3 ± 2.8
	1.0 ± 0.1	46.2 ± 2.1
	2.0 ± 0.1	78.0 ± 2.0

Nano-formulation	Power (W/cm^2)	SAR (W/g)
PLGA[AS-48-BMNPs]	0.5 ± 0.1	18.3 ± 2.8
	1.0 ± 0.1	46.2 ± 2.1
	2.0 ± 0.1	78.0 ± 2.0
